# Demography-dependent variability in the human tumor mycobiome

**DOI:** 10.1128/spectrum.02310-25

**Published:** 2026-03-26

**Authors:** Dan Coster, Thomy Margalit, Ronen Ben-Ami, Ben Boursi, Ron Shamir

**Affiliations:** 1 Blavatnik School of Computer Science and AI, Tel-Aviv University, Tel Aviv, Israel; 2 Gray Faculty of Medicine and Health Sciences, Tel Aviv University 26745, Tel Aviv, Israel; 3 Department of Infectious Diseases, Tel Aviv Sourasky Medical Center 26738, Tel Aviv, Israel; 4 Department of Oncology, Sheba Medical Center 26744, Ramat-Gan, Israel; 5 Center for Clinical Epidemiology and Biostatistics, University of Pennsylvania 6572, Philadelphia, Pennsylvania, USA; Nova Southeastern University, Fort Lauderdale, Florida, USA

**Keywords:** mycobiome, microbiome, tumor, cancer

## Abstract

Recent studies have shown that the tumor mycobiome may have prognostic and diagnostic significance in cancer patients. We aimed to gain a better understanding of how patient characteristics (age, sex, body mass index [BMI], and race) influence the composition of the tumor mycobiome, using the data of these studies. We first tested the data in view of recent critiques of tumor microbiome data processing procedures and concluded that the batch correction and transformation used on it may produce false signals. Instead, we explored 14 combinations of data transformation and batch correction methods on data of 224 fungal species across 13 cancer types. Propensity scores were utilized to adjust for potential confounders such as histological type and tumor stage. To minimize false outcomes, we identified as positive results only those fungi species that showed significant difference in abundance across a demographic factor within a particular cancer type, using data normalized according to all 14 combinations. We observed significant differences in 24 fungal species abundance within tumors for certain demographic characteristics. A total of 20 of these differences were among races in specific cancers. The findings indicate that there are intricate interactions between the mycobiome, cancer type, and patient demographics. Our study highlights the need to account for race in order to understand the role of the mycobiome in cancer development and treatment response. The study also underscores the importance of data processing techniques.

**IMPORTANCE:**

This study analyzes the demographic-dependent variability of the intratumor mycobiome, providing a novel understanding of fungal abundance across different cancer types and patient demographics. By analyzing over 5,000 tumor samples from The Cancer Genome Atlas, the research identified 24 fungal species with significant abundance variations linked to demographic factors such as race, age, sex, and body mass index. These findings underscore the complexity of the tumor microenvironment and the importance of accounting for demographic diversity in cancer research. The study emphasizes the necessity of using robust data normalization and batch correction techniques to avoid spurious associations in order to ensure the reliability of mycobiome analysis. This work highlights the mycobiome as a new frontier in precision oncology and paves the way for future personalized cancer diagnostics and treatments that account for the influence of demographic factors on tumor biology.

## INTRODUCTION

The role of the mycobiome, the fungal component of the microbiome, in tumorigenesis has gained appreciation in recent years (1). Fungi, such as *Malassezia globosa* and *Aspergillus rambellii*, are involved in the development of pancreatic ductal adenocarcinoma (PDAC; see Table S4 for cancer name abbreviations) and colorectal cancer (CRC), respectively, through varied mechanisms, such as the secretion of bioactive metabolites and modulation of host immunity (2–5). Moreover, fungi show promise as a possible target for cancer treatment and prevention. Fungal dysbiosis has been linked to CRC, and modulation of the mycobiome was suggested as a possible prevention strategy (6). In addition, ablation of the mycobiome was reported to be protective against tumor growth in mice PDAC models (4).

While the impact of demographic and clinical factors on the composition of the bacterial and viral components of the tumor microbiome has been thoroughly investigated (7–9), little is known about their association with the composition of the cancer mycobiome. A single multicohort analysis in CRC patients showed a variation of gut fungal communities according to the geography, sex, and disease stage (5). However, to the best of our knowledge, the effect of demographic factors on the variability of the cancer mycobiome has not been analyzed systematically to date. The gut mycobiome is highly dynamic, more so than the bacterial microbiome, and varies by population age, sex, dietary habits, ethnicity, and geography, as well as in many disease states and their associated treatments (10). The majority of studies assessing the cancer mycobiome have been conducted on relatively small and homogenous sample populations (11, 12). This underscores the need for a comprehensive exploration of the impact of various factors to ensure the validity of conclusions drawn from such analyses (11, 12). Comprehending the ramifications of these factors can improve disease understanding and treatment.

Recently, Narunsky-Haziza et al. characterized the tumor mycobiome in 17,401 tumor samples covering 35 cancer types using The Cancer Genome Atlas (TCGA) data (13). This work followed an extensive study of bacterial and viral microbiomes across various cancer types in the same data set by Poore et al. (14). We wished to study the demographic mycobiome variability using the data of 13. However, that study used some of the data-processing methods employed by Poore et al., which were subsequently criticized (15) and recently retracted. Briefly, three methodological problems were identified: insufficient filtering of human reads, identification of bacterial genera as significant despite having no previous records of existence in humans, and artificial signals resulting from improper data transformation and batch-correction process.

Our goal was to assess associations between intratumoral fungal abundance and patient characteristics, using the RNA-Seq mycobiome data of Narunsky-Haziza et al. We first re-evaluated the data in view of the methodological critiques. We show that one of the three methodological problems persisted and propose a methodology to rectify it. This was achieved by applying multiple batch correction and normalization procedures. Then, we identified associations between various demographic factors and the abundance of fungi within the tumor. To mitigate potential confounding effects and enhance the robustness of our analyses, we employed both propensity scores and Inverse Probability of Treatment Weighting (IPTW) (16, 17). This framework allowed us to identify demographic-associated differences in tumor-linked fungal abundance with increased confidence.

## MATERIALS AND METHODS

### Data

**Fig 1:**
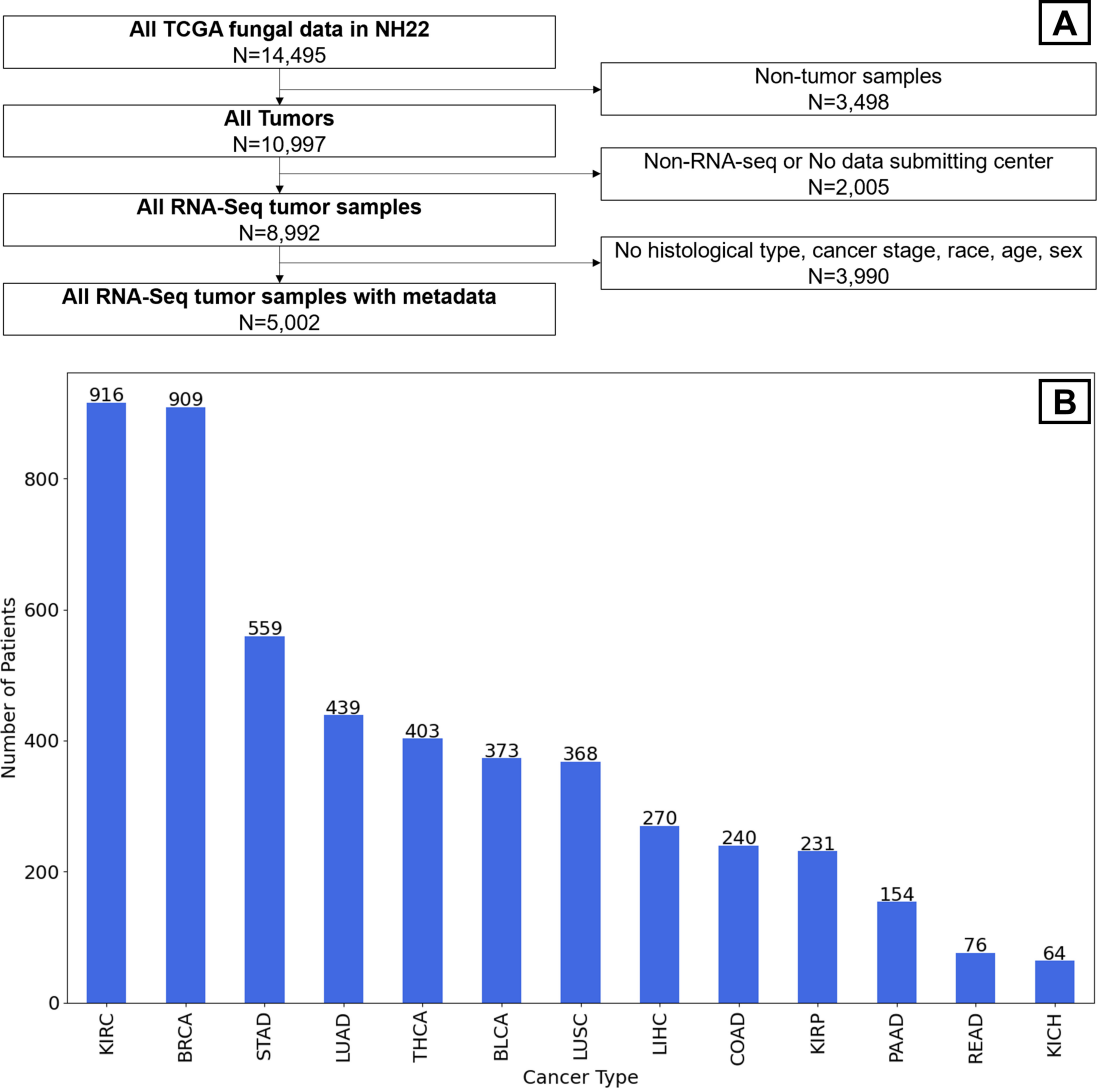
(**A**) Study design. (**B**) The number of analyzed samples of each cancer type. The Cancer Genome Atlas (TCGA) cancer name abbreviations are available in Table S4.

We acquired normalized and read raw counts of intratumor fungal abundance, categorized by species, from 14,495 individual samples originating from TCGA (18). Samples were labeled by cancer type, sex, age at diagnosis, and race, encompassing a total of 32 distinct cancer types. The data set was available from Narunsky-Haziza et al. (NH22) (detailed information provided in Supplementary information 1). We focused exclusively on 5,002 RNA-seq samples that were categorized as primary tumors (Fig. 1A). Additionally, body mass index (BMI) values were extracted for a subset of the samples from Hu et al. (19).

### NH22 data processing

NH22 employed a multistep pipeline for the retrieval of raw counts of metagenomic data. Briefly, reads deemed human or viral, too short, or of poor quality were filtered out. The remaining were categorized by microbial species and underwent an additional decontamination step. Reads that mapped to a microbial reference genome were tabulated as hits. Results were summarized in a table where samples constituted the rows and microbial genome IDs the columns, containing the abundance of each species in each sample. This Decontaminated Fungal Raw Count (NH22-DFRC) table served as the basis for subsequent downstream analyses. Two additional processing steps were applied on that data set: data transformation using *Voom (*20) (log-CPM and quantile normalization) and batch correction using *snm (*21). This produced the Decontaminated Fungal Normalized data matrix (NH22-DFN, Supplementary information 1).

### Critique of data processing techniques

NH22 used some of the same data processing methods previously employed by Poore et al. (P20), which were subsequently criticized by Gihawi et al. (G23). Three main concerns were raised: (i) A considerable fraction of the genomic reads initially categorized as bacterial were, in fact, human. This occurred due to inaccuracies in the genomic database and the computational methodologies employed. (ii) The data contained artificial signals, where bacterial genera with almost no reads in the data were deemed important for cancer classification. This happened due to the transformation methods, which were based on inappropriate statistical assumptions. (iii) Some genera identified by the models as important had no previous records of existence within the human microbiome. The latter two issues were also highlighted in an earlier study (22). While the original study by Poore et al. was later retracted, the analytical approach was refined in the authors' subsequent study of NH22, and the authors argued that the aforementioned concerns have been adequately addressed (23, 24).

### Cohort enhancement in NH22

Narunsky-Haziza et al. expanded and improved the data set in multiple ways: They added data from 1,183 tumor and normal tissues, which underwent additional procedures for detection of fungal contamination (Weizman Institute of Science [WIS] data set). The TCGA data were reprocessed for better alignment and computational decontamination, and fungi species found were filtered against those found in WIS, in the Human Microbiome Projects' gut mycobiome cohort (25) and in a literature review of 100+ publications. Ultimately, this process led to the exclusion of 95 fungal species as potential contaminants, while retaining 224 species as bona fide noncontaminants in the data set. In particular, this process alleviated the third concern as only fungi previously reported in humans were included in the analysis.

### Testing the number of microbial reads

We wished to test if the critique on the inflated number of microbial reads was true on the NH22 data. Gihawi et al. studied in detail three TCGA cancer types: bladder urothelial carcinoma (BLCA, *n* = 683), head and neck squamous cell carcinoma (HNSC, *n* = 334), and breast invasive carcinoma (BRCA, *n* = 238). They re-analyzed these data sets using a stricter pipeline, thus producing novel and lower bacterial raw counts for these cancer types (referred to as G23-BRC). To test the critique, we compared bacterial raw counts from NH22, P20, and G23; see Supplementary information 1. To ensure an equitable comparison, only those samples and bacterial genera common to G23-BRC, NH22-BRC, and P20-BRC were analyzed, yielding data from 716 samples (BRCA, *n* = 232, HNSC, *n* = 330, and BLCA, *n* = 154), spanning 155 genera. Results were compared by Pearson’s correlation, and *P*-values were derived by Student’s *t*-test for correlation and the Mann-Whitney (MW) test. Furthermore, to overcome possible sample size differences across samples, we computed the abundance ratio between pairs of genera within each sample and calculated the correlation of these pairwise ratios between samples. To avoid dependency between different ratios, we randomly subsampled sets of 77 disjoint genus pairs (covering together 154 distinct genera) and computed the correlation of their ratios. This process was repeated 2,000 times, obtaining a distribution of correlation values for each data set.

### Testing the effect of normalization

G23 argued that the data transformation and batch-correction procedures applied in P20 (Voom-SNM) generated the reported artificial signals. Since NH22 employed the same normalization methods, we wished to assess whether a similar artificial signal was present in that data set. We focused on fungi species that were among the 10 most important features in the classifiers built in NH22 for distinguishing between tumor and normal samples in cancer types showing good classification (AUPR > 0.8 and AUROC > 0.9) (Supplementary information 1). We sought such species with a raw count of zero for more than 95% of the samples, reasoning that their contribution to the classification task should be negligible. We also compared the values of these species between the raw counts (NH22-DFRC) and the normalized data set (NH22-DFN).

### Alternative normalizations and batch correction

We used several batch-correction and transformation methods on the NH22 data instead of the Voom-SNM normalization. Methods for data transformation included the following: Centered-Log Ratio (26) (CLR) (with 0 values offset to 1) and CLR_C (27), a variant of CLR that imputes 0 counts, relative abundance, and *Voom (*20). Voom was utilized after quantile normalization as in NH22 and P20. To correct for potential batch effects caused by different sequencing centers, we tested ComBat (28), Batch Mean Centering (BMC) (29), MMUPHin (30), and PLSDA (31). RBE (32) produced equivalent results to BMC in our tests, so we did not include it. We used only unsupervised normalization and batch-correction methods to minimize the risk of bias or signal leakage as supervised methods have previously been suspected to introduce such artifacts (15).

In our analysis, we tested all combinations of transformation and batch correction methods listed above. It is noteworthy that MMUPHin specifically accepts raw counts or relative abundance counts as input and produces the same types of data as output. Therefore, we first applied MMUPHin to our data and then the data transformation. In all other cases, the transformation preceded batch correction. Additionally, since PLSDA can be used only after CLR transformation, we used it only with CLR and CLR_C. In total, we applied 14 combinations.

### Fungal differential abundance

We gathered tumor and patient characteristics from the TCGA data set. Tumor factors included tumor stage (1–4) and histological type, and patient attributes included age at diagnosis, sex, and race (defined as African, European, and Asian).

We evaluated five independent variables: sex, age, (i.e., age ≥70 vs age <70), and pairwise race comparisons: European vs Asian, European vs African, and Asian vs African. Our dependent variables were the fungal species. To mitigate potential confounding effects, we employed a two-step approach. First, we computed propensity scores using logistic regression based on the independent variables. Subsequently, we applied the IPTW scheme, as performed in 16, 17. We performed one-hot encoding of categorical variables and categorized tumor stage into four levels (1, 2, 3, and 4) to facilitate the analysis (for example, 3A, 3B, and 3C were considered as 3). This procedure was instrumental in correcting confounding bias. We ensured that all the potential confounders were well-balanced between the groups, with standardized differences of less than 10%, in line with previous research (17, 33). For each transformed and batch-corrected fungal species, we fitted a linear regression model with the IPTW weights and computed the model’s *P*-value. We corrected for multiple hypotheses using Bonferroni’s method. Species with *P*-value < 0.05 were deemed statistically significant. Notably, when evaluating the effect of a patient characteristic on fungal species, we excluded it from the IPTW calculation. Thus, we repeated the IPTW procedure for each independent variable and for each cancer type with 20 or more samples in each of two groups.

BMI was available for a subset of the samples (*N* = 669, 13.4%). We examined its influence (binarized into obese [BMI > 30] vs non-obese [BMI ≤ 30] categories), considering all other patient characteristics (age at diagnosis, sex, and race) as potential confounders in IPTW. All data processing and statistical analyses were carried out using R statistical software version 4.2.0 and the Python programming language 3.4.

## RESULTS

We obtained data of 14,495 samples from the TCGA and following the application of exclusion criteria (Methods) were left with 5,002 samples of tumors that had RNA-Seq data, information on the sequencing center, and patient characteristics (Fig. 1A). The number of samples per cancer type varied between 916 in kidney renal clear cell carcinoma (KIRC) and 64 in kidney chromophobe (KICH) (Fig. 1B). The distribution of demographic characteristics varied across cancer types. For example, female percentage was 99% in BRCA vs 54% in lung adenocarcinoma (LUAD) samples. In all cancer types, the most prevalent race was European. The distribution of the age, BMI, tumor stage, and histological type also varied between cancer types (Table S1).

**Fig 2:**
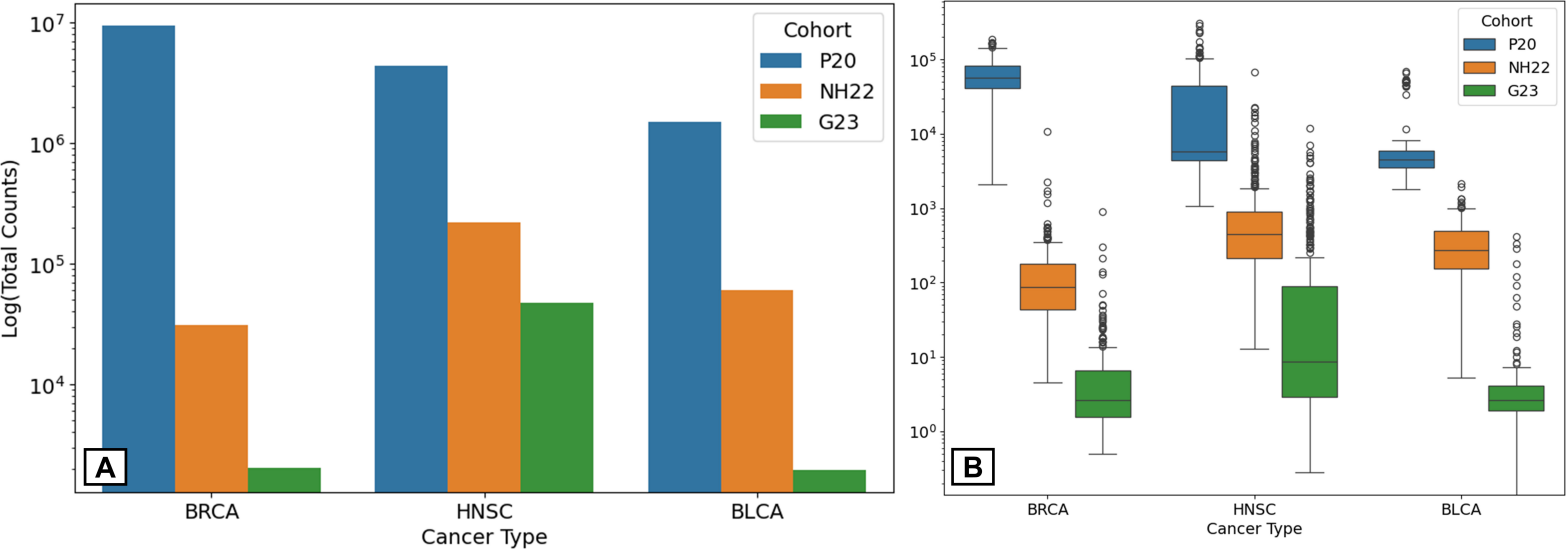
Statistics on the count of bacterial reads after filtering in the P20, NH22, and G23 data sets. (**A**) Total count. (**B**) Boxplots depicting the distribution of the counts per sample.

**Fig 3:**
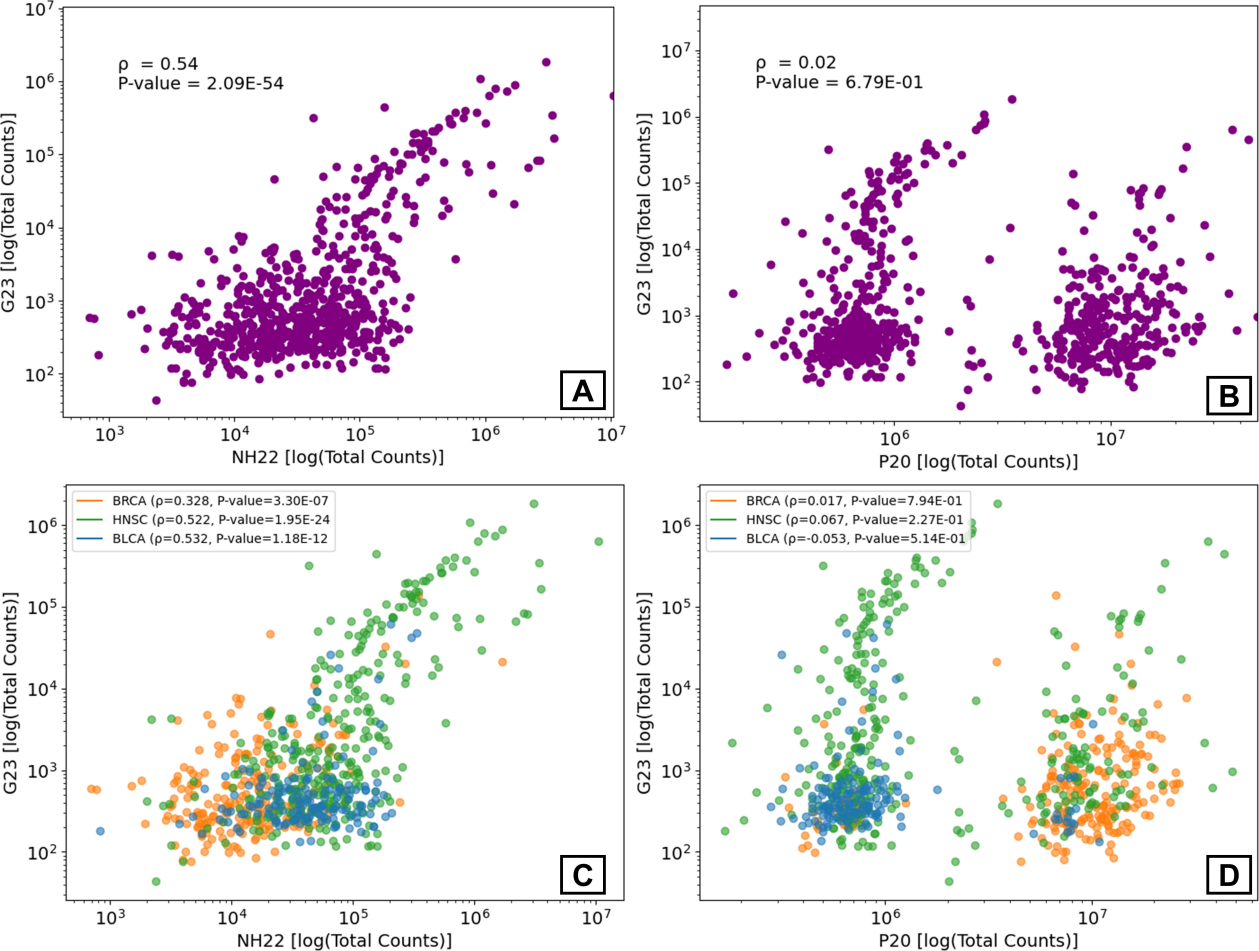
Correlation plots of the total bacterial read count per sample. (**A**) G23 vs NH22. (**B**) G23 vs P20. (**C**) G23 vs NH22, analyzed separately for each cancer type. (**D**) G23 vs P20, analyzed separately for each cancer type. Each point corresponds to a sample.

Recently, Gihawi et al. (G23) raised concerns regarding the validity of the analysis of microbiome data in the TCGA by Poore et al. (14) (P20). As NH22 used some of the same preprocessing methods, we first wished to ensure the validity of the NH22 data (see Materials and Methods for further details). One concern was inflated microbial read counts. To check this, we compared the data of NH22, P20, and the subset of data of three cancer types that was re-analyzed in G23. To ensure an equitable comparison, only samples and bacterial genera common to G23, NH22, and P20 were analyzed, yielding 716 samples (BRCA, *n* = 232; HNSC, *n* = 330; and BLCA, *n* = 154) spanning 155 genera. The total number of bacterial reads in NH22 was 2–3 orders of magnitude lower than those in P20 and 1–2 orders of magnitude higher than those in G23 (Fig. 2A). Similar ratios were noted in the distributions of the bacterial read counts per sample (Fig. 2B). Thus, the differences between NH22 and G23 are indeed less significant than those between P20 and G23. Furthermore, the read count per sample of G23 showed a stronger correlation with NH22 than with P20 (r=0.54 vs r=0.02, Fig. 3A and B). Similar trends were observed when each cancer type was evaluated separately (Fig. 3C and D).

Next, we compared the bacterial data of NH22 and G23 at the genus level. The total number of reads per genus was correlated (r=0.67, *P* = 4.17E-21, Fig. S1A), and this correlation persisted in two of three cancer types when analyzed separately (BRCA: r=0.323, *P* = 4.01E-05; HNSC: r=0.757, *P* = 2.83E-30; BLCA: r=0.025, *P* = 0.075, Fig. S1C). P20 was far less correlated to G23 in this analysis (Fig. S1B and D).

As an additional test that avoids potential differences in the total read counts among the data sets, we computed the ratios between read counts of different genera within each data set (pairwise ratios) and then calculated the correlation between these ratios in NH22 and G23, and in P20 and G23 (taking steps to avoid dependency, see Materials and Methods). The correlation of NH22 to G23 was significantly higher than that of P20 to G23 (*P* = 5.59E-132, Fig. S2). Similar results were observed per cancer type (results not shown).

**Fig 4:**
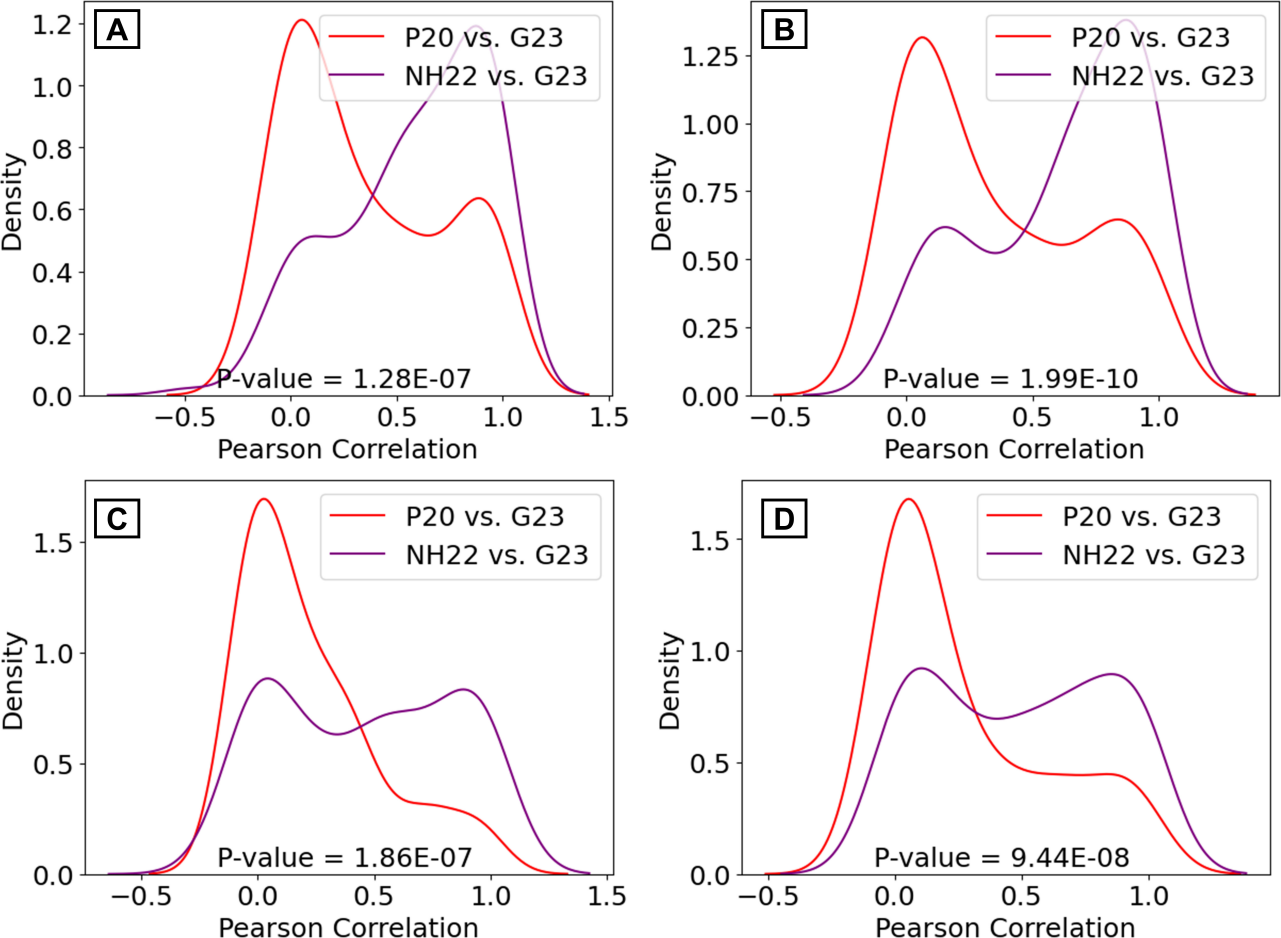
Distribution of the correlation between genera read counts across samples. The distribution includes the values for all genera. Each figure shows the correlations for NH22 vs G23 and P20 vs G23. (**A**) All cancers. (**B**) HNSC. (**C**) BLCA. (**D**) BRCA.

Next, for each bacterial genus and cohort, we computed the correlation between the genus’ read counts across samples and plotted the distribution of the correlation values across the genera. The correlations between NH22 and G23 were significantly higher than those between P20 and G23 (Fig. 4; Table S2). The same was true when we separately analyzed the five most prevalent genera in G23 - defined as those with the highest number of samples with no zero read counts (Fig. S3): *Pseudomonas* (NH22 vs G23; r=0.684, *P* = 1.2E-99, P20 vs G23; r=0.02, *P* = 0.6), *Staphylococcus* (NH22 vs G23; r=0.565, *P* = 2.7E-50, P20 vs G23; r=-0.155, *P* = 3.3E-05), *Acinetobacter* (NH2 vs G23; r=0.197, *P* = 1.1E-06, P20 vs G23; r=-0.02, *P* = 0.6), *Corynebacterium* (NH22 vs G23; r=0.959, *P* = 8.8E-306, P20 vs G23; r=0.957, *P* = 3.3E-313), and *Klebsiella* (NH22 vs G23; r=0.944, *P* = 1.4E-171, P20 vs G23; r=0.017, *P* = 0.7). Similar results were obtained when repeating this analysis per cancer type (Fig. S4 to S6 and Table S2). Overall, the analysis results showed that the issue of inflated bacterial read count in P20 raised by G23 was addressed to a large extent in NH22 and that many characteristics of the NH22 data were similar to that in G23.

**Fig 5:**
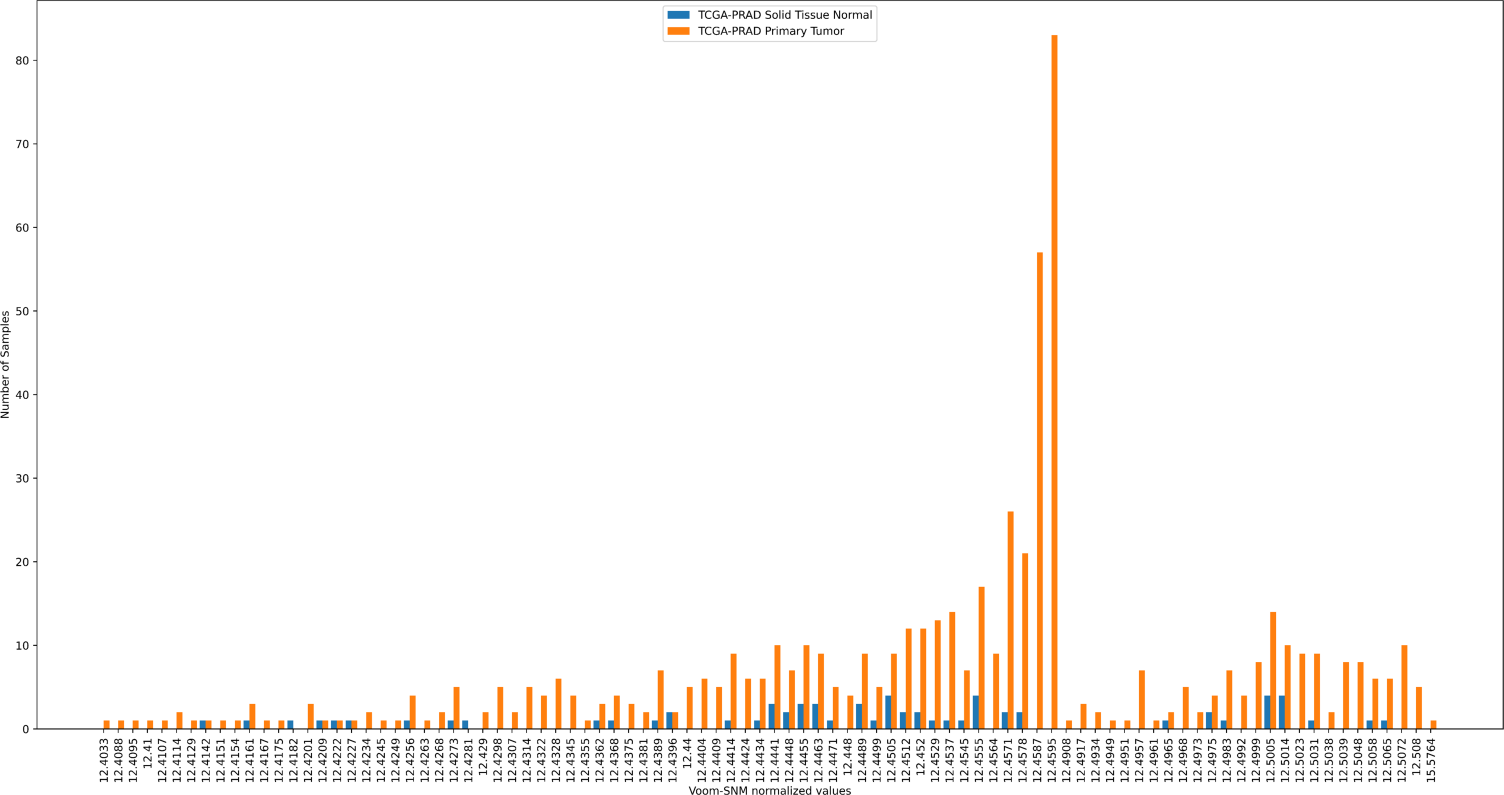
The effect of Voom-SNM normalization. Distribution of normalized counts of *Parastagonospora* for prostate adenocarcinoma (PRAD) primary tumor samples (blue) vs normal tissue samples (orange). Before the normalization, out of 666 samples, 665 had 0 count and 1 had a count of 1.

Another important issue raised by G23 in P20 was the procedure used to remove batch effects and convert the raw counts to normalized values. P20 used Voom-SNM (20, 21) for that purpose. Cancer type classifiers built in P20 using the processed data identified bacterial genera as important features, even though they were absent in most or all samples. Since Voom-SNM was also used in NH22, we wished to test if fungi-based classifiers built by NH22 also suffered from the same problem. We examined the ten genera with the highest feature importance used in the primary tumor vs normal tissue classifier and found some examples of this phenomenon:

*Parastagonospora* was the third-most important feature for the classifier in prostate adenocarcinoma (PRAD). Out of 666 PRAD samples of primary tumor and normal tissue (of both RNA-seq and whole-genome sequencing [WGS]), 665 had 0 *Parastagonospora* reads, and only 1 had 1 read. As illustrated in Fig. 5, the extremely nonrandom and right-skewed distribution of the primary tumor normalized values—all of which except 1 started as raw values of 0—makes it easy for a machine learning classifier to separate the PRAD primary tumor samples from normal solid tissue samples. Based on that distribution, a model that splits the samples using the threshold 12.457 labels 332/351 of the positive samples correctly (PPV = 94.6%), with a relatively high sensitivity of 55.33%. *Ramularia* was the sixth-most important feature for the classifier in KIRP. Out of 345 KIRP samples, 344 had 0 *Ramularia* reads, and 1 had 1 read. The distributions of the normalized values are extremely different (Fig. S7). A threshold of 17.36 obtains PPV = 95.6% with a sensitivity of 49.84%. We conclude that following Voom-SNM normalization, false signals persist in the NH22-processed data.

**Fig 6:**
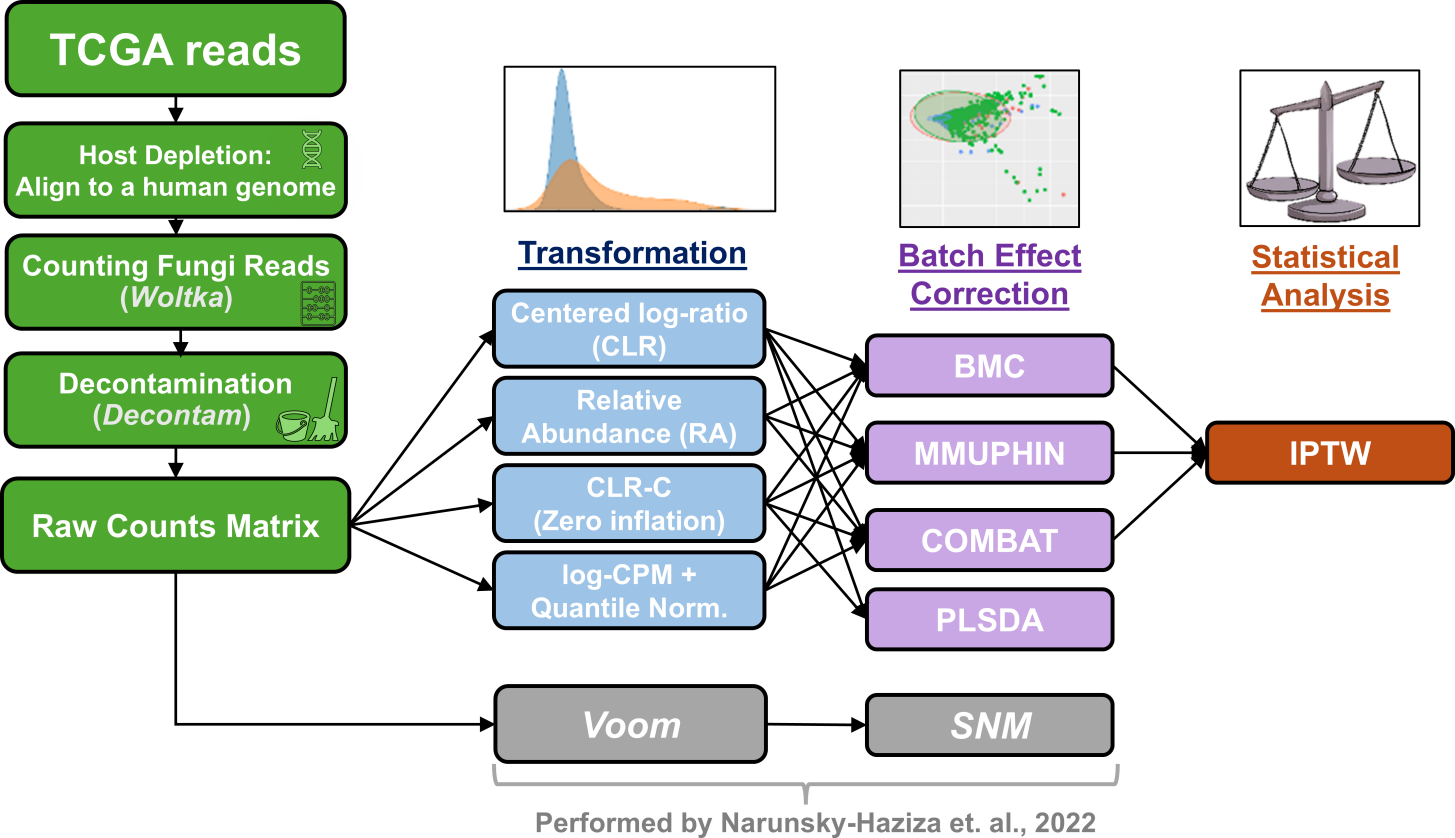
The pipeline used to clean and normalize the fungi data. The central part shows the combinations of transformation and batch correction used.

Our main question in this study was whether fungi species abundance varies in cancer patients with different demographic characteristics. Batch correction and data normalization are key for the answer, so instead of Voom-SNM, we used 14 combinations of normalization and batch correction approaches (Fig. 6 and Materials and Methods). NH22 used SNM to correct two types of batch errors: specimen type (RNA-seq or DNA) and the sequencing center of origin. Since most current batch correction methods were designed to correct one type of error, we decided to use only the RNA-Seq samples since they cover most of the cohort (8,992/10,997 tumor samples). We used propensity score and IPTW for correction of potential confounders: age, sex, race, tumor stage, and histological type (Methods). We applied IPTW separately to each demographic factor and then fitted weighted linear regression for each fungal species, using the IPTW weights. The *P*-values from this analysis were corrected for multiple hypotheses using Bonferroni’s method. To err on the side of caution, we sought species that were significant in all 14 combinations. A total of 24 species showed consistent statistically significant differences between race groups (Table S3). We note that race in this analysis functions as a broad proxy for multiple socioenvironmental, cultural, and genetic factors and should not be interpreted as a direct biological determinant.

In the comparison between European vs. African, there were five significant fungal species in colon adenocarcinoma (COAD): *Candida orthopsilosis, Penicillium expansum, Diutina rugosa, Aspergillus alliaceus, and Aspergillus pseudonomius*; two species in LUAD: *Diutina rugosa and Aspergillus pseudotamarii*; and two species in lung squamous cell carcinoma (LUSC): *Candida orthopsilosis and Aspergillus welwitschiae*. In the comparison between European vs Asian, there were nine significant fungal species in stomach adenocarcinoma (STAD): *Aspergillus clavatus, Aspergillus terreus, Aspergillus aculeatus, Penicillium digitatum, Pestalotiopsis fici, Penicillium expansum, Aspergillus novofumigatus, Aspergillus campestris, and Ramularia collo-cygni* and one species, *Metarhizium robertsii,* in liver hepatocellular carcinoma (LIHC). In the comparison of Asian vs African populations, one species, *Candida auris,* was found in thyroid cancer (THCA). In comparison of males vs females, one species, *Purpureocillium lilacinum,* was found in STAD. In comparison of BMI ≥30 vs BMI < 30, one species, *Diutina rugosa,* was found in LIHC. In comparison of elderly (age > 70) vs young (≤70), one species, *Hyphopichia burtonii,* was found in LIHC, and one species, *Aspergillus oryzae,* was found in BRCA. We also examined raw count distributions for the significant fungi species. Notably, 23 of the 24 species remained significant even without normalization or adjustments (Table 1). Finally, to ensure that extreme IPTW weights did not influence the results, we repeated the analysis after truncating weights at the 99th percentile. The findings were identical to those obtained using the full weights.

**TABLE 1:** The combinations of fungus, cancer, and demographic factor that passed the analysis pipeline and their statistics^*a*^

Species	Cancer	Factor	Group 1				Group 2				*P*-Value
			Variable	*N*	Mean ± Std	%	Variable	*N*	Mean ± Std	%	
*Candida orthopsilosis*	COAD	Race	European	179	2.034 ± 2.934	75.4	African	49	1.204 ± 3.298	38.8	6.44E-06
*Candida orthopsilosis*	LUSC	Race	European	332	0.666 ± 0.865	48.8	African	28	0.107 ± 0.315	10.7	1.08E-04
*Penicillium expansum*	COAD	Race	European	179	1.419 ± 2.038	69.3	African	49	0.796 ± 2.354	24.5	5.41E-07
*Aspergillus welwitschiae*	LUSC	Race	European	332	1.801 ± 1.804	81	African	28	0.643 ± 0.951	42.9	1.98E-05
*Diutina rugosa*	COAD	Race	European	179	3.05 ± 4.18	85.5	African	49	1.673 ± 4.446	44.9	4.01E-07
*Diutina rugosa*	LUAD	Race	European	383	2.486 ± 4.448	67.6	African	47	1.298 ± 4.348	29.8	4.10E-06
*Aspergillus alliaceus*	COAD	Race	European	179	2.011 ± 2.804	72.6	African	49	1.163 ± 3.294	30.6	1.75E-06
*Aspergillus pseudotamarii*	LUAD	Race	European	383	3.958 ± 6.829	80.4	African	47	2.234 ± 7.29	42.6	9.41E-07
*Aspergillus pseudonomiae*	COAD	Race	European	179	8.603 ± 11.607	92.7	African	49	5.122 ± 13.579	63.3	2.70E-08
*Aspergillus clavatus*	STAD	Race	European	383	2.885 ± 26.302	37.9	Asian	159	4.943 ± 14.467	61	4.72E-08
*Aspergillus terreus*	STAD	Race	European	383	1.368 ± 7.03	32.9	Asian	159	4.377 ± 27.276	47.2	5.74E-04
*Metarhizium robertsii*	LIHC	Race	European	140	0.364 ± 0.691	27.1	Asian	116	0.103 ± 0.382	7.8	8.27E-05
*Penicillium digitatum*	STAD	Race	European	383	2.439 ± 8.56	42.8	Asian	159	5.541 ± 18.228	57.9	2.18E-04
*Pestalotiopsis fici*	STAD	Race	European	383	1.371 ± 11.924	26.6	Asian	159	4.0 ± 16.497	43.4	4.52E-05
*Penicillium expansum*	STAD	Race	European	383	2.525 ± 7.963	47.3	Asian	159	7.226 ± 17.541	55.4	1.99E-03
*Aspergillus aculeatus*	STAD	Race	European	383	0.982 ± 8.263	21.2	Asian	159	1.786 ± 5.704	36.5	9.96E-05
*Aspergillus novofumigatus*	STAD	Race	European	383	3.295 ± 30.454	39.2	Asian	159	5.774 ± 19.619	54.1	5.63E-05
*Aspergillus campestris*	STAD	Race	European	383	2.815 ± 24.615	40.5	Asian	159	4.39 ± 14.698	47.8	8.19E-03
*Ramularia collo-cygni^b^*	STAD	Race	European	383	57.277 ± 221.833	77.3	Asian	159	14.308 ± 31.794	58.5	4.02E-07
*Candida auris*	THCA	Race	African	23	0.261 ± 0.541	21.7	Asian	51	1.118 ± 1.259	62.8	9.50E-04
*Purpureocillium lilacinum*	STAD	Gender	Male	353	0.334 ± 0.899	17	Female	206	0.17 ± 0.659	9.7	1.43E-02
*Aspergillus oryzae*	BRCA	Age	>70	169	0.432 ± 0.807	30.2	≤70	740	0.22 ± 0.588	16.9	5.74E-05
*Hyphopichia burtonii*	LIHC	Age	>70	53	0.453 ± 0.867	32.1	≤70	217	0.101 ± 0.384	7.8	2.20E-06
*Trichoderma atroviride*	BLCA	BMI	>30	74	0.108 ± 0.354	9.5	≤30	299	0.064 ± 0.327	4.7	1.14E-01

^*a*^ The combinations that passed the pipeline were those that were significant in all 14 normalization and batch-correction methods. *N*, number of samples in the group. Mean ± Std, mean and standard deviation of raw read counts per sample in the group. %, fraction of samples that had at least 1 read of the fungus.

^*b*^ Ramularia colloo-cyngi is a likely contamination. See (ref). *P*-values were calculated using the Mann-Whitney test, on the raw fungal counts. COAD, colon adenocarcinoma; LUAD, lung adenocarcinoma; LUSC, lung squamous cell carcinoma; STAD, stomach adenocarcinoma; LIHC, liver hepatocellular carcinoma; THCA, thyroid cancer.

## DISCUSSION

In this study, we examined how different demographic factors relate to fungal abundance across multiple tumor types, by analyzing a data set comprising over 5,000 tumor samples from the TCGA. We observed that the abundance of several fungal species in particular cancer types differed significantly based on patient demographics, with the most pronounced differences noted between various racial groups. These patterns mirror what have been observed in bacterial tumor microbiome studies and suggest that similar principles may also apply to fungi. Considering demographic diversity may therefore be important when interpreting tumor-associated microbial and fungal data and when developing future hypotheses on the host-microbe interaction in cancer.

In our analysis, we took several steps to address recent concerns raised about microbial analyses in cancer. We developed a pipeline that utilizes a consensus of multiple methods for batch correction and data normalization and also utilized propensity scores when calling the association between a particular fungus and a particular demographic factor in a specific cancer. Additionally, our results were supported by the raw (un-normalized) RNA-seq read counts. This increases robustness and reliability of the results and reduces the risk of spurious associations. Some of our main findings were as follows: We observed distinct differences in *Candida orthopsilosis* abundance between European and African populations within samples of COAD and LUSC. Additionally, variations in *Candida auris* abundance were observed between African and Asian populations within samples of THCA. Previous research has shown a close relationship between *Candida* species and tumors of the colon and stomach (34). Specifically, intestinal *Candida* has been associated with the development of hepatic and gastric carcinogenesis (35, 36). We also identified significant variations in the abundance of various *Aspergillus* species between European and African populations in samples of COAD, LUSC, and LUAD. Furthermore, we noted differences between European and Asian populations in samples of STAD. A previous study has indicated an association between *Aspergillus rambellii* in the gut mycobiome and CRC (5). The study accounted for several potential confounding factors such as age, sex, BMI, and tumor location, but it did not assess the potential impact of race.

Ethnicity-related variation in the mycobiome may reflect dietary and geoclimatic factors. For example, the abundance of *Candida* spp. in the gut correlates with a Western-type carbohydrate-rich diet and is negatively associated with a protein- or fatty acid-rich diet (37). In contrast, the mycobiome of vegetarians is dominated by environmental spore-forming fungi, such as *Fusarium*, *Penicillium,* and *Aspergillus* spp (38). Geography was also shown to exert an effect on the gut mycobiome. Spore-forming filamentous fungi, including *Aspergillus* spp. and *Penicillium* spp., are enriched in hot and arid climates and rural environments. In a study from China, geographical location and ethnicity-related dietary habits were the major identified drivers of gut mycobiome composition (39). Importantly, ethnicity and dietary-related mycobiome patterns correlate with gastrointestinal inflammation and may be associated with cancer risk either directly or through aberrant immune responses (10). For example, expansion of *Candida* spp. in the gut has been linked to both gastrointestinal inflammation and translocation of *Candida*-derived β-glucan and candidalysin into the bloodstream, leading to inflammation in extraintestinal organs (10). Moreover, elevated *Candida* burden in colorectal cancer, which we found to be associated with European ethnicity, was shown to induce glycolysis in macrophages and trigger IL-22 production by innate lymphoid cells (40). Members of the *Aspergillus flavus* species complex, which we identified in association with European ethnicity in colorectal cancer (*A. alliaceus*) and lung adenocarcinoma (*A. pseudotamarii*), produce the carcinogenic mycotoxins ochrotoxin A and aflatoxin, respectively (41).

These patterns reinforce the view of the tumor as an ecological niche, where environmental exposures, immune context, and demographic-associated factors can shape fungal presence. We emphasize that demographic categories like race serve as multifaceted surrogates of socioenvironmental, cultural, and genetic influences. Consequently, observed associations should be viewed through this integrative lens rather than as evidence of direct biological causation.

Recently, Ge et al. (42) improved the computational analysis of Gihawi et al. (15) and proposed some fungi species that are a likely contamination. The species *Ramularia collo-cygni*, which was found by our analysis, is likely a vector contamination, as noted by Ge et al. Furthermore, Ge et al. (42) shared a data file of cancer samples and their WGS read counts of the fungi species. Unfortunately, that sample size was too small to enable validation of our results.

The tumor mycobiome has been studied across pancreatic (3, 4), lung (43), esophageal (44), oral (45), and renal (46) cancers. Together with recent reviews (47–49), these studies open diagnostic, prognostic, and therapeutic opportunities for fungal characterization and underscore the broader importance of understanding how fungal signals vary across patient groups. Our analysis provides additional granularity by demonstrating that these fungal signals vary across demographic strata. Recognizing such variability will be essential for designing clinically relevant microbial biomarkers and understanding interpatient heterogeneity.

Our study contributes to our understanding of the intricate relationships between demographic factors, mainly race, and the intratumor mycobiome. It also emphasizes the importance of incorporating demographic diversity into cancer research and clinical practice. By accounting for the influence of race and other demographic variables on the tumor microenvironment, future studies may develop more personalized and effective strategies for cancer prevention, diagnosis, and treatment and propose novel therapeutic interventions targeting the tumor mycobiome. Such advances have the potential to ultimately improve patient outcomes and enhance precision cancer medicine.

## Data Availability

The open-source Python and R code used for the computational analysis is available at: https://github.com/Shamir-Lab/FungiCancer. The results presented here are in whole or part based on data generated by the TCGA Research Network: https://www.cancer.gov/tcga. The STORMS checklist for this manuscript is available at https://zenodo.org/records/15774962.

## SUPPLEMENTAL MATERIAL

Supplemental materials spectrum.02310-25-s0001.docx Supplemental information and Figures S1 to S7.

Table S1 spectrum.02310-25-s0002.csv Cohort characteristics of different cancer types. The top five most common values of Histological Type and Tissue Source Label are presented for each cancer type.

Table S2 spectrum.02310-25-s0003.csv Pearson correlation between the counts of each species in NH22-BRC vs. G23-BRC and P20-BRC vs. G23-BRC in all cancers and per cancer type.

Table S3 spectrum.02310-25-s0004.xlsx The number of fungal species that were identified as statistically significant across all 14 combinations of normalization and batch correction per each comparison.

Table S4 spectrum.02310-25-s0005.xlsx TCGA study name abbreviations.

ASM does not own the copyrights to Supplemental Material that may be linked to, or accessed through, an article. The authors have granted ASM a non-exclusive, world-wide license to publish the Supplemental Material files. Please contact the corresponding author directly for reuse.
